# Sex differences in neuropsychiatric symptoms in mild cognitive impairment

**DOI:** 10.1002/alz70857_107013

**Published:** 2025-12-26

**Authors:** Rashmina Shrestha, Cheryl Cheuk Yan Y Leung, Latha Velayudhan

**Affiliations:** ^1^ Oxleas NHS Foundation Trust, London, United Kingdom; ^2^ Institute of Psychiatry, Psychology and Neuroscience, King's College London, London, United Kingdom

## Abstract

**Background:**

Neuropsychiatric symptoms (NPS) in mild cognitive impairment (MCI), an intermediate state between normal aging and dementia, is gradually gaining in interest. Sex differences have been studied in Alzheimer's dementia but but there is limited information on sex differences in NPS in MCI. We aimed to study sex differences in prevalence of individual symptoms and severity of NPS in people above 65 years with MCI.

**Method:**

This study included participants with ICD‐10 diagnosis of MCI recruited from memory services within South London and Maudsley National Health Trust, UK (Research Ethics Committee reference number: 06/Q0706/50). They underwent clinical assessments including Consortium to Establish a Registry for Alzheimer's Disease, Mini mental state examination (MMSE), olfactory identification test, and Neuropsychiatry Inventory‐ Clinician version (NPI‐C) for NPS.

**Results:**

46 participants (mean age 79.22 years ±7.12; 59 % women) had mean MMSE score of 25.72 (±2.93) were evaluated for NPS. Both groups were similar in their MMSE scores (*p* = 0.28). Men had significantly higher prevalence of aberrant vocalizations (*p* =  0.03). Men exhibited significantly higher severity of irritability (*p* = 0.10) and dysphoria (*p* = 0.05) compared to women. Details of individual NPS prevalence and relation to other clinical and sociodemographic factors will be further presented.

**Conclusion:**

This study underscores sex differences in NPS prevalence and severity in MCI, highlighting the importance of more research to understand the pathophysiology and their role in risk prediction to dementia progression.

